# Child Life under Queen Victoria

**Published:** 1897-03-27

**Authors:** 


### Child Life under Queen Victoria.

### VII.—Merely Players.

Hitherto we have spoken of children who, if not consciously aggrieved, were consciously suffering. Even when the plea of economic necessity was raised to prevent their wrongs being righted, humanitarian sentiments spoke strongly on the children's side. But we now come to cases in which the humanitarian necessity for intervention was less obvious, but not less real.

Until very recently one of the most popular, and certainly one of the prettiest parts of every pantomime, and of many other theatrical spectacles, was a ballet or procession of children. Whole opera companies of children toured the country, and in other plays some child-performer was often the chief attraction. These children were, as a rule, well fed, kindly-treated, and liked their work; many people thought it unnecessary to interfere with them, many even thought it unkind. Yet, if one thinks for a little, it is easy to see how unwholesome such a life must be.

These children were often apprenticed to the ballet-masters or others who were to train them for the stage for a term of nine years. The apprenticeship began when the child was very young—five years of age, or sometimes four. During the whole time of the apprenticeship the child was at the absolute disposal of the trainer, who, when she—the vast majority of the children were girls—had learned enough, accepted engagements for her not only in London, but in the provinces. Troupes of trained child-dancers have even been sent abroad. Naturally, the teachers liked to keep the children as busy as possible. Their health might not be neglected, but their education was, and they were made to feel that their dancing was the main object of their lives. Children who were not apprenticed, but whose parents exploited them, were no better off than their neighbours; the parent who is supported by his child is often the severest taskmaster. And, indeed, where a whole troupe went from one school more care would be taken for their safety in travelling and comfortable accommodation than would be bestowed on a single child.

But, on the face of it, stage-life is bad for a child. The performance takes place at night, and it may well be that a child who does not live near the theatre would not get home until after midnight. In the case of pantomimes there were often morning performances twice a week; the double performance was a heavy strain on the energies of young children. There were also rehearsals to fatigue the child. It might be thought that the requirements of compulsory education would at least prevent a child from performing during the day, but absence from school for one afternoon in the week would not of itself hinder anyone from making up the requisite number of attendances, and Saturday, of course, was a school holiday.

As to rehearsals, theatre-managers would considerately (?) arrange to have them during the dinner-hour. The little dancer would therefore have a long day of mental and physical work, with hardly time to snatch a hasty meal, and in the evening a performance for which only an unwholesome excitement made them fit. Such a double strain is obviously bad. Moreover, the atmosphere and temperature of a theatre are too often unhealthy. Dressed in flimsy gauze, the child was exposed to draughts, perhaps when over-heated with exertion or with posing in the hot "flies." Nor can the moral effects of the life be forgotten. Precocious self-sufficiency is almost inevitable in juvenile performers—the self-importance of being a bread-winner, often accentuated by being the recipient of some extra dainty or bit of finery as a reward; while a premature acquaintance with the evil that is in the world is a thing that must too often have taken place.

When any attempt is made to interfere with child-labour, in one form or another, there is sure to be an outcry that the little wage-earner is the sole support of some aged and infirm relative. Even when this plea is true, it is no excuse for the continuance of an evil. The future must not be sacrificed to the past, and it is better that the aged grandmother should accept parish help than that the grandchild's health and education should suffer, as with these stage-children it did. And in many cases there was no special need for the children to work. They were not drawn from the poorest home—the children of the very poor are not plump and fresh enough to make good stage-pictures. But their parents were sometimes greedy, oftener idle and extravagant, and the child's earnings proved a convenient addition to the family exchequer.

So far we have spoken of children who were the victims of no wilful cruelty, but there were cases where as much could not be said. It can hardly be denied that cruelty is frequent in the training of acrobats and circus-riders; indeed, cases have been proved in a court of law of the ill-treatment that marks the training for such work. It is only if it is begun when the muscles are flexible that the results can be successful, and trainers declare that when commenced early enough in life it is not painful. Even this may be doubted; but it is certain that even when there is no pain there is fear. The feats which a gaping audience trembles and wonders at must seem terrible to a little child, and only the fear of punishment will make it attempt them. And it is admitted that this dread is by no means unfounded. Punishment, and the fear of punishment, are the original inducements to learn these feats which, when learned—so much we may admit—give pleasure and gratification to the performer as well as the spectators.

If anyone of reasonable age chooses to become acrobat or stage-player let them be free to do so; but the contention on which all legal action for children is based is that they are not free agents in choosing their work, and must, therefore, be protected from being put to any work that is dangerous or unwholesome. On this ground the legislature has interfered, at least to a limited extent, with child performers, by forbidding children under the age of eleven being allowed to perform in a place of public amusement. A special licence for a child-player may, however, be granted. With regard to the circus-performers, a fine of £25 is imposed on anyone who allows a child under the age of sixteen to be trained as acrobat or contortionist, unless in the case of a parent or legal guardian who trains the child himself. Even this concession may occasionally bear hardly on the child; it will certainly tend to create a hereditary caste of acrobats.

